# A large palindromic segmental duplication on mouse chromosome 12 marks an evolutionarily fragile genomic interval

**DOI:** 10.1186/s12864-026-13210-y

**Published:** 2026-07-27

**Authors:** Yong-Kook Kang

**Affiliations:** Aging Convergence Research Center (ACRC), Aging Research Center, Korea Research Institute of Bioscience Biotechnology (KRIBB), 125 Gwahak-ro, Yuseong-gu, Daejeon, 34141 South Korea

**Keywords:** Segmental duplication, Palindromic duplication, Inversion, Mouse chromosome 12, CpG island, Genome structure, Synteny comparison

## Abstract

**Background:**

Palindromic segmental duplications (SDs), in which duplicated sequence arms flank a central spacer in inverted orientation, are among the most structurally consequential configurations in mammalian genomes, yet their occurrence in the mouse genome remains poorly characterized. Here, SD12A1 is systematically characterized as a large palindromic SD locus on mouse chromosome 12 (chr12:17.95–24.51 Mb, mm10).

**Results:**

Analysis of 9,263 UCSC mm10 pairwise SD entries, integrated with de novo CpG island (CGI) profiling, directional sequence matching, block-level sequence identity analysis, and mouse–rat–human synteny comparison, showed that SD12A1 comprises two SD-rich arms flanking a 530 kb SD-depleted central gap. Inter-arm SD pairs were predominantly inverted, whereas intra-arm pairs were predominantly direct. Reverse-complement CGI analysis identified 18 left-arm CGIs with high-confidence right-arm matches (best-match RC cosine similarity > 0.75), and directional matching of 50 bp Block 1 queries showed forward hits enriched in the left arm and reverse-complement hits enriched in the right arm. Cross-arm sequence identity was broadly uniform (median 93.0%; median Jukes–Cantor distance = 0.0730), with nearly identical within-arm and cross-arm divergence distributions (median JC 0.0747 vs. 0.0735), providing no evidence for ongoing inter-arm gene conversion. Two block pairs showed substantially higher sequence identity than the main palindrome bulk (B03<-> B17, 98.6%; B06<-> B20, 97.1%), suggesting more recent localized duplication. Three-species synteny comparison identified a 7.1 Mb orientation-discordant interval, shared by mouse and rat relative to human, that encompasses SD12A1 and corresponds to a rearrangement-prone human orthologous region.

**Conclusions:**

These findings establish SD12A1 as a mouse-specific palindromic manifestation of an evolutionarily labile genomic interval and provide a reference-based framework for recognizing large autosomal palindromic SD architecture in mammalian genomes.

**Supplementary Information:**

The online version contains supplementary material available at https://doi.org/10.1186/s12864-026-13210-y.

## Background

Segmental duplications (SDs) — blocks of near-identical sequence (≥ 90% identity, ≥ 1 kb) present at two or more genomic locations — account for approximately 5–7% of the human genome and represent one of the most dynamic sources of structural variation in mammalian genomes [1–3]. Their importance extends beyond genome assembly complexity: SDs are the primary substrate for non-allelic homologous recombination (NAHR), which generates recurrent deletions, duplications, and inversions at dozens of disease-associated loci, including DiGeorge syndrome (22q11.2) [4], Williams–Beuren syndrome (7q11.23) [5], and Charcot–Marie–Tooth disease (17p12) [6]. However, not all SDs pose equivalent structural risk. The rearrangement potential of a duplicated region depends critically on the spatial and orientational relationship between paralogous copies: directly oriented repeats favor deletions and duplications via intrastrand misalignment, whereas inverted palindromic SD pairs create substrates for inversion and for the formation of hairpin intermediates that can precipitate more complex rearrangements [4, 7].

Palindromic SD architectures — in which two arms of duplicated sequence flank a central unique spacer in inverted orientation — are among the most structurally consequential configurations in the mammalian genome. The paradigmatic example is the human Y chromosome, whose eight large palindromes (P1–P8) maintain arm-to-arm identity exceeding 99.9% through ongoing inter-arm sequence homogenization, and whose spacers harbor dose-sensitive genes essential for male fertility [8]. Yet palindromic SD organization is not confined to the Y chromosome. On autosomes, palindromic core duplicons at chromosome 15q13.3 (*GOLGA8* repeats) have been shown to demarcate the breakpoints of recurrent microdeletions associated with intellectual disability, schizophrenia, and epilepsy, and to have driven multiple independent inversion events during primate evolution [9]. Similarly, palindromic AT-rich repeats at 22q11.2 underlie the most common microdeletion syndrome in humans, with de novo rearrangements detectable in normal sperm at measurable frequency [4, 10, 11]. A shared feature of these loci is that the palindromic architecture itself — rather than any specific gene content — constitutes the structural predisposition to rearrangement, by providing inverted repeat substrates for NAHR-mediated inversion and fold-back replication errors. Whether similar palindromic SD structures exist at comparable scale in the mouse genome, and what their properties and evolutionary origins might be, remains poorly understood.

Several mouse SD loci have been subjected to locus-scale structural analysis, and in each case the dominant organizational principle has been tandem rather than palindromic: the *R2d* locus (chr2:83–84 Mb, mm10) shows strain-specific tandem amplification of intronless retrogene-derived copies [12]; the intelectin (*Itln*) locus (chr1:171–172 Mb, mm10) shows copy-number polymorphism driven by tandem repeat variation [13]; and the pseudoautosomal region (PAR, chrX:169–170 Mb, mm10) contains lineage-specific structural variants organized as tandem duplications [14]. Mouse chromosome 12 has itself been identified as one of the chromosomes with the greatest enrichment of large SD blocks in the murine genome [15], placing it in a broader context of duplication-prone chromosomal architecture, yet its internal SD organization has not been examined at equivalent structural resolution. The ~ 17.5–24.5 Mb interval of chr12 (mm10) is particularly striking in this regard: the strand-orientation pattern of SD records in the UCSC mm10 genomicSuperDups track is suggestive of a bipartite inverted arrangement, raising the specific hypothesis that this region may constitute a large-scale autosomal palindromic SD structure — a configuration not previously described at this scale in the mouse genome, and organizationally distinct from all characterized mouse SD loci to date.

The structural interest of this interval extends beyond the mouse genome. The orthologous region in humans — mapping to chromosome 2p25.1–25.3 — is the site of recurrent, familial inverted duplications, with breakpoints mapping to simple repeat sequences at the interval boundaries [16–18]. The recurrence of pathogenic rearrangements at the human ortholog, combined with the palindrome-suggestive SD strand pattern in mouse, points to a genomic interval that is structurally labile across mammalian lineages — and one for which systematic structural characterization in the mouse reference genome is currently lacking.

This study presents a systematic structural characterization of this locus, designated SD12A1. Strand-orientation profiling, de novo CpG island analysis, UCSC mm10 segmental duplication database interrogation, and three-species synteny comparison together demonstrate that SD12A1 forms a large inverted palindrome composed of two SD-rich arms flanking a central gap. The analyses further show that the palindromic organization is mouse-lineage-specific, that the two arms share a common duplication ancestry without evidence of subsequent inter-arm sequence homogenization, and that the locus resides within a broader orientation-discordant interval—consistent with its classification as an evolutionarily labile genomic region.

## Methods

### Gene annotation of SD12A1

All genomic analyses were performed in R using Bioconductor and tidyverse packages unless otherwise specified. Gene content within SD12A1 (chr12:17,950,503–24,510,004, mm10) was retrieved from the UCSC knownGene annotation using the Bioconductor package TxDb.Mmusculus.UCSC.mm10.knownGene. Gene ranges were obtained with GenomicFeatures::genes(), and genes overlapping the SD12A1 interval were extracted using GenomicRanges::subsetByOverlaps() with ignore.strand = TRUE. Gene symbols were mapped from Entrez IDs using mapIds() from org.Mm.eg.db. The reported gene count therefore refers to Entrez-mapped UCSC knownGene loci rather than to all transcript isoforms or predicted/noncoding models present in comprehensive GENCODE browser tracks. The gap region was defined as chr12:20,990,000–21,523,000, anchored at the left boundary by the *Asap2* transcription start site and at the right boundary by the start of the first right-arm segmental duplication block.

### Segmental duplication structure analysis

The UCSC mm10 segmental duplication BED file was loaded using data.table::fread(). Pairwise SD entries with source coordinates within SD12A1 were extracted. Overlapping entries were merged into discrete duplicated segments using the reduce() function from GenomicRanges, yielding 22 merged blocks. Block arm assignment (left / gap / right) was determined by position relative to GAP_L (20,990,000) and GAP_R (21,523,000). For tile-based density analysis, SD12A1 was divided into 10 kb non-overlapping tiles using GRanges/IRanges, and the number of overlapping SD entries per tile was counted using countOverlaps(). Mean density was computed separately for the left arm, right arm, and gap region. For source fragment coverage analysis per partner bin, intrachromosomal SD pairs were binned at 500 kb resolution based on partner midpoint position. For each bin, source intervals were merged using reduce() and the number of non-redundant source fragments and total coverage (kb) were computed. For visualization of Block 1 internal structure, all pairwise SD entries with source coordinates falling within Block 1 (chr12:17,952,169–18,089,000) were extracted from the full chr12 SD entry table. Entries were annotated by arm based on strand (+ → Left arm (direct); − → Right arm (inverted)) and plotted as geom_rect() elements ordered by entry index. Partner locations were visualized as lollipop scatter plots. Partner location maps for all 22 merged blocks were produced using the same procedure applied iteratively across gr_segdup_merged. All analyses and visualizations were performed in R using ggplot2 and patchwork.

### Pairwise 6-mer sequence similarity

De novo CpG islands within SD12A1 were identified by sliding a 200 bp window across the SD12A1 sequence (extracted using getSeq() from BSgenome.Mmusculus.UCSC.mm10) in 500 bp steps, retaining windows satisfying GC content > = 50% and observed-to-expected CpG ratio > = 0.60 [19]. Adjacent passing windows within 1,500 bp were merged and regions > = 200 bp were retained as de novo CGIs. For each of the 143 de novo CGIs within SD12A1, a 700 nt sequence window centered on the CGI midpoint was extracted using getSeq(). CGIs were mapped to the 22 merged SD blocks using findOverlaps() from GenomicRanges. All overlapping 6-mers were enumerated using substring(), excluding those containing ambiguous bases (N). 6-mer counts were tabulated and L2-normalized (divided by the Euclidean norm) to produce unit-length frequency vectors. Pairwise cosine similarity between all CGI pairs was computed as the matrix inner product using tcrossprod().

### Self-alignment dot plot

To visualize the pairwise alignment structure of SD12A1 in a format analogous to StainedGlass-style self-alignment plots [20], all intrachromosomal SD entries with both source and partner coordinates within the SD12A1 interval (chr12:17,950,503–24,510,004) were extracted from the UCSC mm10 genomicSuperDups track. Each entry was drawn as a line segment connecting its source start–end coordinates (x-axis) to its partner start–end coordinates (y-axis). Segments were colored by strand orientation and sequence identity: direct entries (strand = ‘+’) were rendered in blue and inverted entries (strand = ‘−‘) in red, with color intensity scaled to fracMatch value (range 90–100%) using a gradient from light to dark within each orientation class. The visualization was generated using ggplot2 with the ggnewscale package to apply independent color scales for direct and inverted entries. Unlike StainedGlass, which operates on raw pairwise alignments derived from long-read assemblies across a broad identity range, the present implementation is based on pre-filtered genomicSuperDups annotation and is therefore constrained to the ≥ 90% identity range of that track.

### Strand-based intrachromosomal SD pair analysis

The UCSC segmental duplication BED file was re-read with six named columns (chr, start, end, partner, score, strand) using data.table::fread(). For each SD entry with source coordinates within SD12A1, the partner chromosome and start position were parsed from the partner field (format: “chr: position”). Analysis was restricted to intrachromosomal pairs in which both source and partner midpoints fell within SD12A1. Source midpoint (src_mb) and partner midpoint (partner_mb) were computed in Mb units. A source-partner scatter plot was generated using ggplot2::geom_point().

### Cross-assembly validation (mm39)

To verify that the palindromic architecture of SD12A1 is not specific to the mm10 (GRCm38) assembly, sequence-level comparison was performed between mm10 and mm39 (GRCm39) using chromosome 12 FASTA sequences obtained from the UCSC Genome Browser. The mm39 orthologous interval was anchored by searching a 500 bp probe derived from *Asap2* exon 1 (mm10 chr12:21,269,672–21,270,171) against the full mm39 chr12 sequence using matchPattern() from the Biostrings package (≤ 15 mismatches), yielding a single forward hit at mm39 chr12:21,319,673 (coordinate shift: +50,001 bp). All subsequent mm39 comparisons applied this shift to align mm10 and mm39 coordinates. Tile-by-tile sequence identity was computed across the full SD12A1 interval at 1 kb resolution (*n* = 6,559 non-overlapping tiles). For each tile, the mm10 sequence and the SHIFT-corrected mm39 sequence were extracted using subseq(), and the number of mismatched positions was quantified using neditAt() from the Biostrings package. Percentage identity per tile was computed as 100 × (1 − mismatches / tile length). GC content was additionally computed for each of 655 non-overlapping 10 kb tiles using letterFrequency(), and Pearson correlation between mm10 and mm39 GC profiles was calculated using cor(). To verify that the inverted arm relationship between left and right arms is preserved in mm39, eight 100 bp seeds were extracted from confirmed left-arm SD block intervals (source entries with fracMatch ≥ 0.958, source length ≥ 100 kb, and inverted partner coordinates within the right arm) identified from the UCSC mm10 genomicSuperDups track. Each seed was searched against both the mm10 and mm39 SD12A1 intervals in forward and reverse-complement orientations using matchPattern() (≤ 10 mismatches). Hit positions were recorded and compared between assemblies. All analyses were performed in R using the Biostrings, ggplot2, and patchwork packages.

### Sequence-level palindrome confirmation

To test for inverted sequence homology between left-arm and right-arm CGIs, three pairwise similarity metrics were computed across all left × right CGI pairs (71 × 58 = 4,118 pairs). For the forward comparison, 6-mer frequency vectors were constructed for each CGI sequence as described above. For the RC comparison, the reverse complement of each right-arm CGI sequence was obtained using Biostrings::reverseComplement() prior to k-mer enumeration. For the canonical k-mer comparison, each 6-mer was replaced by the lexicographically smaller of itself and its reverse complement, thereby collapsing strand information before similarity computation. In all three cases, frequency vectors were L2-normalized over a shared vocabulary constructed from both left- and right-arm CGI sequences, and cosine similarity was computed as the matrix inner product. Statistical comparison between RC and forward similarity distributions was performed using a paired one-sided t-test (alternative = “greater”) across all 4,118 pairs.

For each left-arm CGI, the best-match right-arm partner (j*) was defined as the right-arm CGI yielding the highest RC-orientation cosine similarity. Forward similarity for each left-arm CGI was then evaluated against the same partner j*, enabling a matched comparison of RC versus forward orientation similarity at a fixed genomic pair. The 18 left-arm CGIs with a best-match RC similarity exceeding 0.75 were designated high-confidence RC-matched pairs. This threshold was selected empirically: the 18 CGIs above 0.75 form a distinct upper-tail cluster in the best-match RC similarity distribution, no CGI falls between RC similarity values of 0.75 and 0.80 such that the number of selected CGIs is unchanged across this interval, and the mean RC–forward similarity gap for selected CGIs (0.562) substantially exceeds that of CGIs falling below the threshold. Lowering the threshold to 0.70 adds five CGIs with a markedly smaller mean RC–forward gap (0.491), suggesting these represent less unambiguous cases. These pairs were visualized as Bezier arc curves connecting left-arm and right-arm CGI midpoints along the chr12 coordinate axis, with arc color scaled to RC similarity and hub right-arm CGIs (matched by ≥ 2 left-arm CGIs) indicated by a circle overlay. The implied palindrome axis for each high-confidence pair was estimated as the midpoint between the left-arm and right-arm CGI genomic positions [(left_mb + right_mb) / 2], and the mean, median, and standard deviation of these estimates were computed across all 18 pairs. Right-arm CGIs serving as the best RC-match partner for two or more left-arm CGIs were designated hub CGIs.

For directional sequence matching, three 50 bp query sequences were extracted from the start, middle, and end of a representative Block 1 repeat unit. Each query sequence and its reverse complement were searched across the full SD12A1 interval, allowing up to five mismatches, corresponding to an approximately 90% local-identity threshold. Match coordinates were classified by arm and strand orientation to test whether forward-orientation hits preferentially mapped to the left arm and reverse-complement hits preferentially mapped to the right arm.

### Pairwise sequence identity, Jukes–Cantor distance, and HKY85 distances

Pairwise sequence identity between segmental duplication entries was obtained from the UCSC Genome Browser Segmental Duplication track (mm10), which provides pre-computed alignment statistics derived from LASTZ/BLASTZ whole-genome pairwise alignments. The fracMatch field [1], representing the fraction of matching bases in the aligned region, was extracted for each SD entry and multiplied by 100 to convert to percentage identity. For each cross-arm block pair, median sequence identity was calculated across all constituent SD entries. Jukes–Cantor (JC) distance [21] was calculated from median pairwise sequence identity using the standard one-parameter correction:

$$\:{d}_{jc}\text{}=-\frac{3}{4}\text{}\mathrm{l}\mathrm{n}(1-\frac{4}{3}\text{}(1-p\left)\right)$$

where *p* is the proportion of differing sites, defined as 1 − fractional identity. This correction accounts for the probability of multiple substitutions at the same site under the assumption of equal substitution rates among all nucleotide pairs. All calculations were performed in R. The Jukes–Cantor model assumes equal substitution rates among all nucleotide pairs and does not account for CpG hypermutability or regional variation in substitution rates; JC distances reported here should therefore be interpreted as relative divergence estimates rather than absolute evolutionary distances. Within-arm divergence was computed as the median JC distance across all SD entry pairs in which both source and partner coordinates fell within the same arm (left–left or right–right). Cross-arm divergence was computed analogously for pairs in which source and partner coordinates fell on opposite arms. The two distributions were compared using a two-sided Wilcoxon rank-sum test to assess whether gene conversion or other homogenizing mechanisms have differentially affected within-arm versus cross-arm sequence identity. Median JC distance was used as the primary summary statistic because SD12A1 contains SD entries with heterogeneous alignment lengths and partially redundant overlaps. As a sensitivity check, length-weighted mean JC distance was also calculated by weighting each pairwise JC distance by its alignment length.

To assess the sensitivity of divergence estimates to substitution model choice, HKY85 distances [22] were additionally computed for all SD12A1 intrachromosomal entries using empirical transition and transversion proportions derived from the transitionsB and transversionsB fields of the UCSC genomicSuperDups track (mean empirical Ti/Tv ratio = 1.128). HKY85 distances were uniformly ~ 1.15× higher than JC distances across all pair types (cross-arm median: 0.0840; within-arm median: 0.0846), with identical rank relationships between within-arm and cross-arm comparisons under both models, confirming that the conclusion of no differential sequence evolution between arms is robust to substitution model choice.

### Genome-wide survey of autosomal inverted SD clusters

To place SD12A1 in a genome-wide context, all intrachromosomal inverted SD entries on mouse autosomes (chr1–chr19) were extracted from the UCSC mm10 genomicSuperDups track using the same criteria applied to define SD12A1: strand = ‘−’ and fracMatch ≥ 0.90. Entries on the same chromosome within 2 Mb of one another were aggregated into a single cluster using GenomicRanges::reduce() with min.gapwidth = 2 × 10⁶. Clusters were retained if they contained ≥ 100 inverted entries, showed an entry density ≥ 100 entries/Mb, and spanned ≥ 2 Mb. chrX and chrY were excluded due to their distinct repeat-enriched organization. Entry density was computed as the number of inverted entries divided by cluster span (entries/Mb). All analyses were performed in R using the GenomicRanges and tidyverse packages.

### Three-species synteny analysis

To determine whether the SD12A1 interval lies within a mouse-specific rearrangement or a broader rodent-shared orientation configuration, transcriptional orientations were compared across orthologous genes in mouse (mm10), rat (rn7), and human (hg38). Orthologous gene relationships and genomic coordinates were compiled for genes mapping to mouse chromosome 12 and their rat and human orthologs. For each orthologous gene comparison, transcriptional orientation was encoded from the annotated strand in each species. Genes were classified as concordant when mouse, rat, and human shared the same transcriptional orientation, and as rodent-shared discordant when mouse and rat shared an orientation that differed from the human ortholog. To precisely localize the boundaries of the orientation-discordant interval, genes were additionally ranked by genomic position in mouse and in human, and the human rank was examined along the mouse gene order to identify the points at which synteny order was disrupted, since strand discordance alone was insufficient to resolve fine-scale boundary placement for genes immediately flanking the interval. This analysis localized the proximal boundary between *Hpcal1* and *Asap2* and the distal boundary between *Rrm2* and *Mboat2*.

## Results

### Segmental duplication density defines a bipartite architecture flanking a central gene-containing gap

**Fig. 1:**
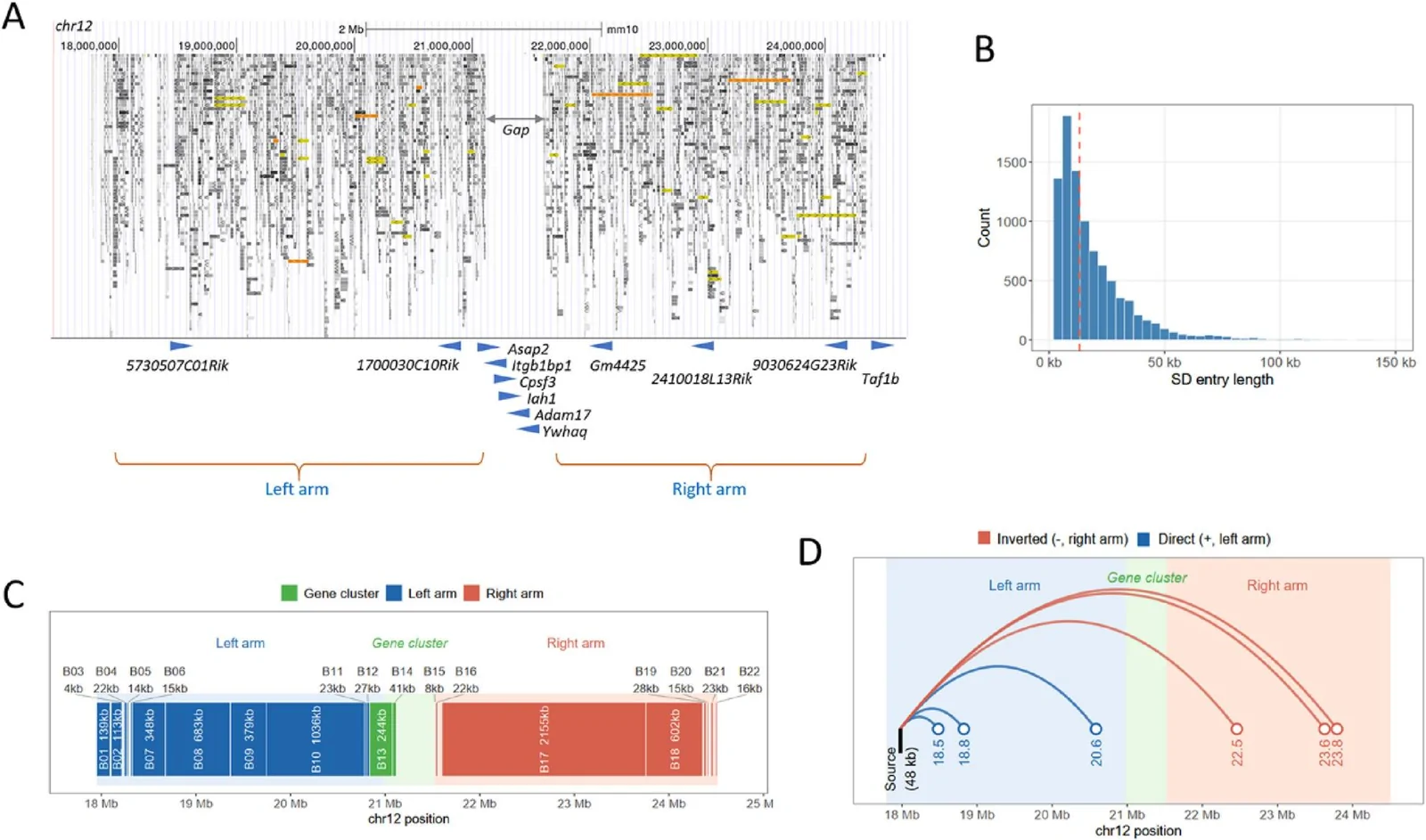
Genomic structure of SD12A1. **A** Overview of the SD12A1 interval on mouse chromosome 12 (mm10). Segmental-duplication entries are shown across chr12:17.95–24.51 Mb, with the left and right SD-rich arms separated by the central SD-depleted gap. Gene labels indicate the 12 Entrez-mapped UCSC knownGene loci overlapping SD12A1: 5730507C01Rik, 1700030C10Rik, Asap2, Itgb1bp1, Cpsf3, Iah1, Adam17, Ywhaq, Gm4425, 2410018L13Rik, 9030624G23Rik, and Taf1b. Comprehensive transcript models are not shown. Blue arrowheads indicate gene transcriptional orientation. **B** Length distribution of the 9,263 pairwise SD entries within SD12A1 (range: 1.4-534.1 kb). The red dashed line indicates the median entry length. **C** Schematic overview of SD12A1 genomic organization. Merged SD blocks (*n* = 22) are shown as colored bars along the chr12 coordinate axis, classified by arm assignment: left arm (blue), gap/gene cluster (green), and right arm (red). Block labels (B1-B22) and sizes (kb) are indicated. **D** Representative pairwise SD entries for a 48 kb source (query) segment at the proximal end of Block 1, illustrating the one-to-many relationship between a single source coordinate and multiple partner (matched) locations distributed across both arms: direct partners (+, left arm; blue circles) and inverted partners (-, right arm; red circles). Each entry in the UCSC genomicSuperDups track is recorded reciprocally—that is, for every source→partner entry, a corresponding partner→source entry exists elsewhere in the database — such that the total entry count reflects pairwise alignments rather than unique physical segments

SD12A1 was defined as chr12:17,952,169–24,510,004 (mm10), corresponding to the outermost coordinates spanned by SD entries within this region. Annotation of genes overlapping this 6.5 Mb interval identified 12 Entrez-mapped UCSC knownGene loci, indicating sparse curated gene content across most of the locus. Six genes—*Asap2*,* Itgb1bp1*,* Cpsf3*,* Iah1*, *Adam17*, and *Ywhaq*—were concentrated within a 427 kb gene cluster at chr12:20,990,459–21,417,637 near the center of SD12A1 (Fig. 1A). Because this gene cluster lies within a broader SD-depleted interval (530 kb) extending to the first right-arm SD block, the central gap was operationally defined as chr12:20.99–21.52 Mb, separating the left and right SD-rich arms.

The UCSC SD track was next examined to define the internal organization of SD12A1. A total of 9,263 pairwise SD entries—each comprising source and partner coordinates representing the query and matched segments of a pairwise alignment—were present within the locus (Fig. 1B). Merging overlapping source coordinates collapsed these entries into 22 blocks ranging from 4.1 kb to 2,155.1 kb (Fig. 1C). A representative 48 kb source fragment from the first segment of Block 1 (chr12:17.95–18.09 Mb, 139 kb) showed multiple partner matches in both arms, providing an initial indication that the merged blocks represent comple × SD-entry clusters rather than simple one-to-one duplicated units (Fig. 1D). Together, these analyses indicate that SD12A1 is composed of two SD-rich arms separated by a central gene-rich gap.

### Merged SD blocks reveal heterogeneous and partially redundant internal organization

**Fig. 2:**
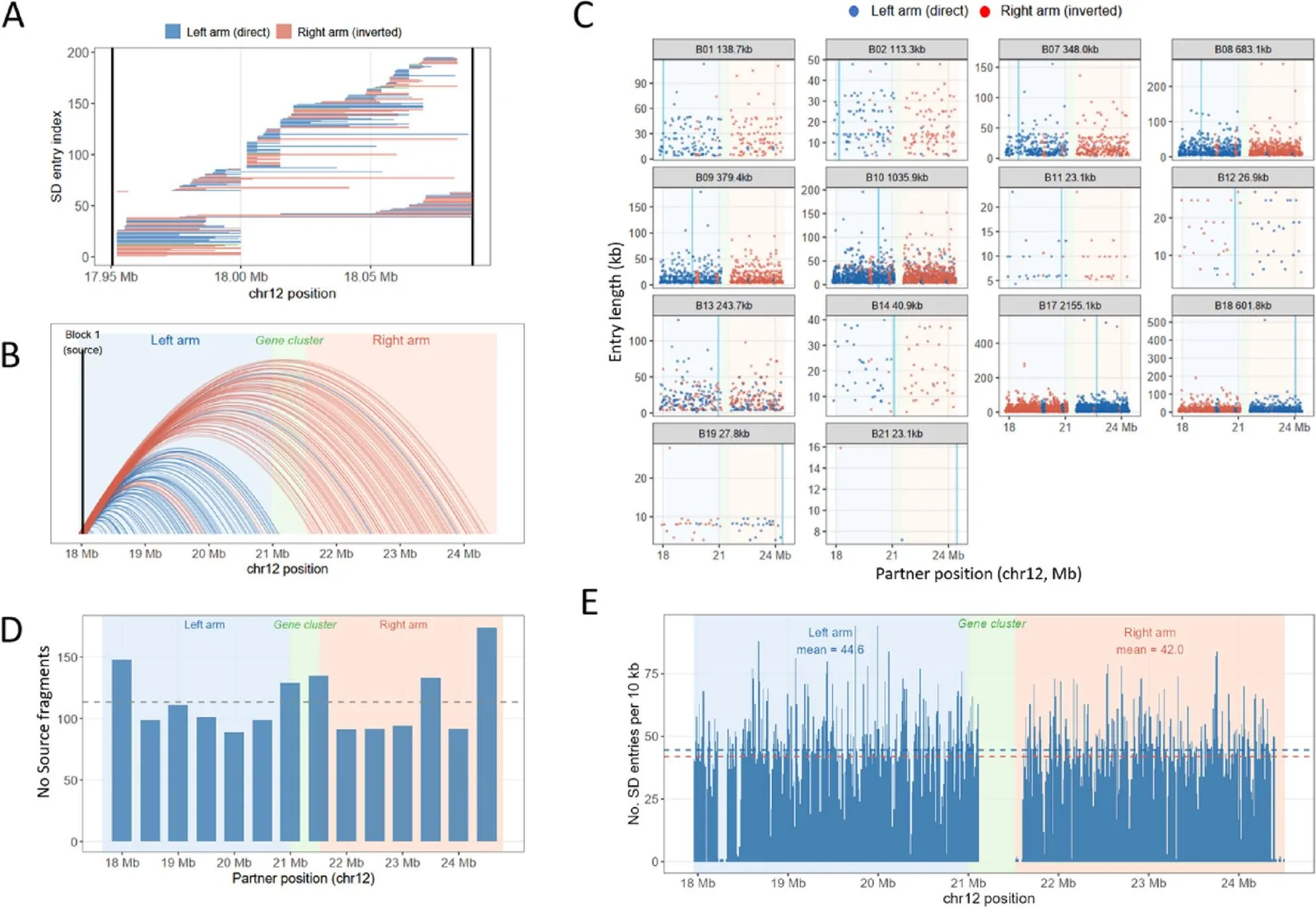
Internal structure and locus-wide connectivity of segmental duplications in SD12A1. **A** Internal entry structure of Block 1 (chr12:17.95–18.09 Mb, 139 kb). Each horizontal bar represents one of the 195 pairwise SD entries contained within Block 1, ordered by entry index (y-axis) and colored by partner arm: blue = left arm (direct, +); red = right arm (inverted, −). **B** Arc diagram of Block 1 pairwise SD relationships. Each arc connects the Block 1 source interval (left boundary, black vertical line) to one partner location along the chr12 coordinate axis. Blue arcs = direct partners in the left arm; red arcs = inverted partners in the right arm. Background shading indicates arm assignment: blue = left arm, red = right arm, green = functional gene gap. **C** Partner location maps for 14 merged SD blocks. Each panel shows the pairwise SD entries contained within one merged block, with partner position along chr12 plotted against entry length. Points are colored by partner arm: blue = left arm (direct); red = right arm (inverted). The light-blue vertical marker indicates the genomic position of the source block shown in each panel. Block labels and total block sizes are shown above each panel. Background shading as in (**B**). **D** Source fragment coverage per 500 kb partner bin. For each 500 kb interval along chr12, the number of non-redundant source fragments targeting that bin is shown. Coverage ranged from 80 to 173 source fragments per bin (mean indicated by dashed line), demonstrating broad locus-wide SD connectivity across both arms and the gap region. **E** SD entry density across SD12A1 in 10 kb non-overlapping tiles. The number of overlapping SD entries per 10 kb tile is plotted along the chr12 coordinate axis. Mean density was 44.6 entries/10 kb in the left arm and 42.0 entries/10 kb in the right arm (dashed line). A pronounced depletion of SD entries over the central gene cluster (green shading) separates the two segmental duplication-rich arms

Although overlapping SD entries were collapsed into 22 merged blocks, these blocks did not generally correspond to simple, uniform repeat units. Block 1, for example, contained 195 pairwise entries distributed across the interval, consistent with an overlapping rather than non-redundant internal structure (Figs. 2A and 2B). Of the 22 merged blocks, 14 were supported by multiple overlapping entries (Fig. 2C). Among the remaining eight single-entry intervals, one represented an interchromosomal alignment with chr5, whereas the others corresponded to reciprocal or boundary representations of existing intra-SD12A1 alignments rather than independent duplication events. Specifically, B06—B20 and B16—B22 formed reciprocal pairs, B04 and B15 both pointed to B21, and B03 corresponded to the start of B17.

Despite this local heterogeneity, duplication connectivity extended across the full locus. Every 500 kb genomic bin was targeted by 80–173 source fragments, indicating broad locus-wide coverage rather than confinement to a few isolated segments (Fig. 2D). Consistently, 10 kb tile-based density profiling revealed high SD density across both arms, with a pronounced depletion over the intervening central region (Fig. 2E). Together, these analyses show that SD12A1 is not a collection of discrete repeat units, but a broadly connected network of overlapping SD-entry clusters distributed across two SD-rich arms. These structural features were independently confirmed in the mm39 (GRCm39) assembly, with 99.6% of 1 kb tiles showing perfect sequence identity between mm10 and mm39 across the locus and concordant GC content profiles (Pearson *r* = 1.000), indicating that the locus architecture is not specific to the mm10 reference assembly used for the primary analyses (Supplementary Fig. S1).

### Strand-orientation analyses support an inverted palindromic architecture of SD12A1 with localized orientation asymmetry

**Fig. 3:**
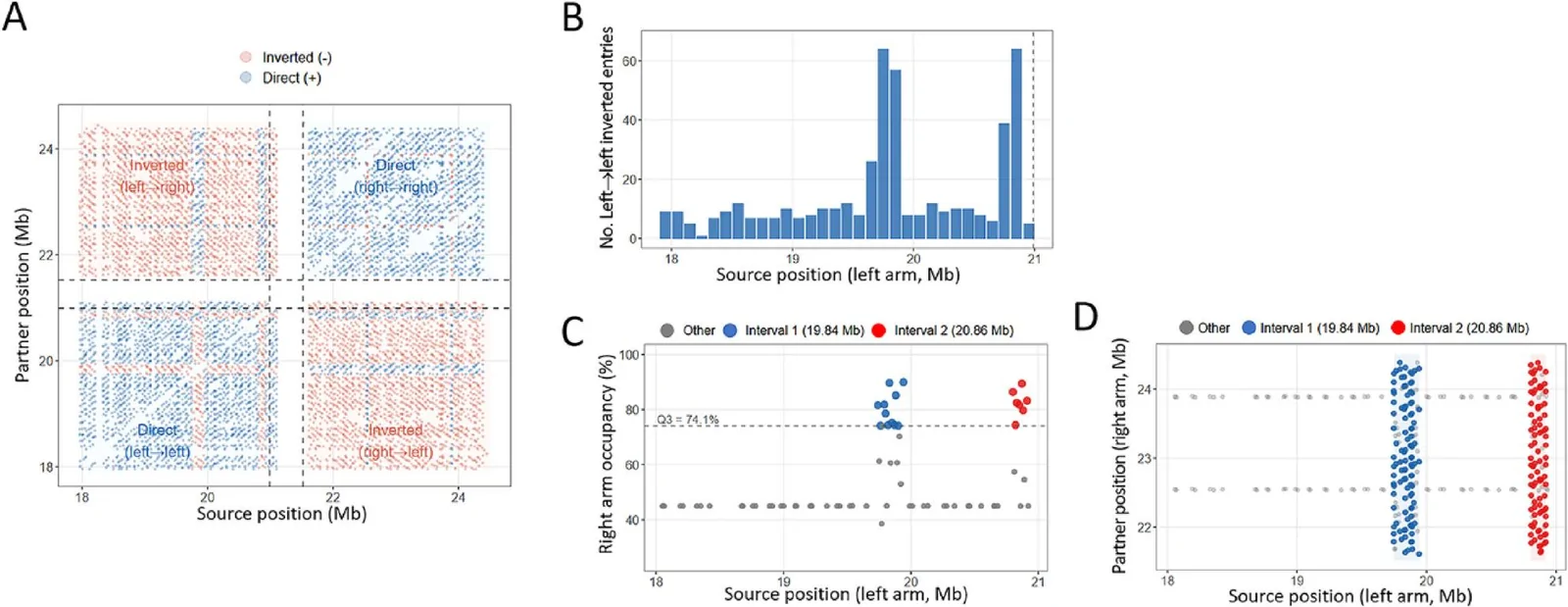
Strand-orientation structure of SD12A1 reveals an inverted palindromic architecture with localized orientation asymmetry. **A** Source–partner scatter plot of intrachromosomal SD pairs within SD12A1. Each point represents one pairwise SD entry, with source midpoint plotted against partner midpoint. Points are colored by orientation: blue = direct (+); red = inverted (−). Dashed lines indicate gap boundaries, dividing the plot into four quadrants. **B** Distribution of intra-arm minority (inverted, −) SD entries along the left arm, binned in 100 kb intervals. Two intervals show disproportionate enrichment: a broad concentration at ~19.75–19.94 Mb and a narrow but intense peak at ~20.81–20.92 Mb, the latter immediately proximal to the left gap boundary (dashed line). **C** Right arm occupancy of left→right direct (+) SD entries, plotted by 20 kb source bins along the left arm. Occupancy is defined as the range of partner positions (maximum minus minimum partner midpoint) within the right arm, expressed relative to total right arm length. This metric reflects the breadth of right-arm correspondence for each source bin, not the density of partner entries. Bins exceeding the third-quartile threshold (Q3 = 74.1%) were classified as high-occupancy. Points are colored by right arm occupancy: blue = high-occupancy interval 1 (19.75–19.94 Mb); red = high-occupancy interval 2 (20.81–20.92 Mb); grey = other left arm intervals. **D** Source (left arm) versus partner (right arm) positions for all left→right direct (+) SD entries. Each point represents one SD entry; source midpoint (x-axis) and partner midpoint (y-axis) are shown in Mb. Points are colored as in panel **C**. This per-entry view confirms that the broad right-arm correspondence observed in panel **C** reflects a genuine distributional pattern across multiple entries, rather than being inflated by isolated outlier partners

The bipartite organization of SD12A1 raised the possibility that the two arms are arranged as inverted counterparts, as expected for a large genomic palindrome [23, 24]. Consistent with this model, plotting source midpoint against partner midpoint for intrachromosomal SD pairs revealed a clear quadrant structure (Fig. 3A). Inter-arm pairs were predominantly inverted, whereas intra-arm pairs were predominantly direct, indicating that the left and right arms are arranged in an inverted relationship around the central gap. A complementary StainedGlass-style dot plot, in which pairwise SD entries were drawn as oriented alignment segments and colored by sequence identity, confirmed the same quadrant organization and arm-to-arm inverted relationship (Supplementary Fig. S2).

Although this overall pattern supports a large-scale inverted palindromic organization, minority-orientation entries were also detected and could mark local substructures within the broader palindrome. In the left arm, intra-arm inverted entries were not uniformly distributed but instead showed two prominent source-position enrichments: one broad interval around 19.75–19.94 Mb and a second, sharper interval around 20.81–20.92 Mb, the latter lying immediately proximal to the left gap boundary (Fig. 3B). Notably, both enriched intervals also showed unusually broad direct-orientation correspondence with the right arm. When left-arm source positions were binned at 20 kb resolution and assessed for right-arm occupancy, only these two intervals consistently exceeded the third-quartile threshold of 74.1%, whereas most other left-arm positions showed much more restricted right-arm coverage (Fig. 3C). Mapping the corresponding partner positions confirmed that direct entries from these two source intervals were distributed across a wide span of the right arm rather than being confined to a localized partner region (Fig. 3D). Together, these results indicate that SD12A1 is organized as an inverted palindrome but is not structurally uniform along its length. Instead, specific subregions within the left arm show disproportionate enrichment of minority-orientation entries and broad direct-orientation correspondence with the right arm. These locally asymmetric orientation patterns indicate internal architectural heterogeneity superimposed on the overall palindromic structure, although the present reference-based analysis does not determine whether these subregions are associated with elevated structural-variation frequency.

### Reverse-complement comparison of cross-arm CGI pairs provides direct sequence-level evidence for inverted cross-arm homology

**Fig. 4:**
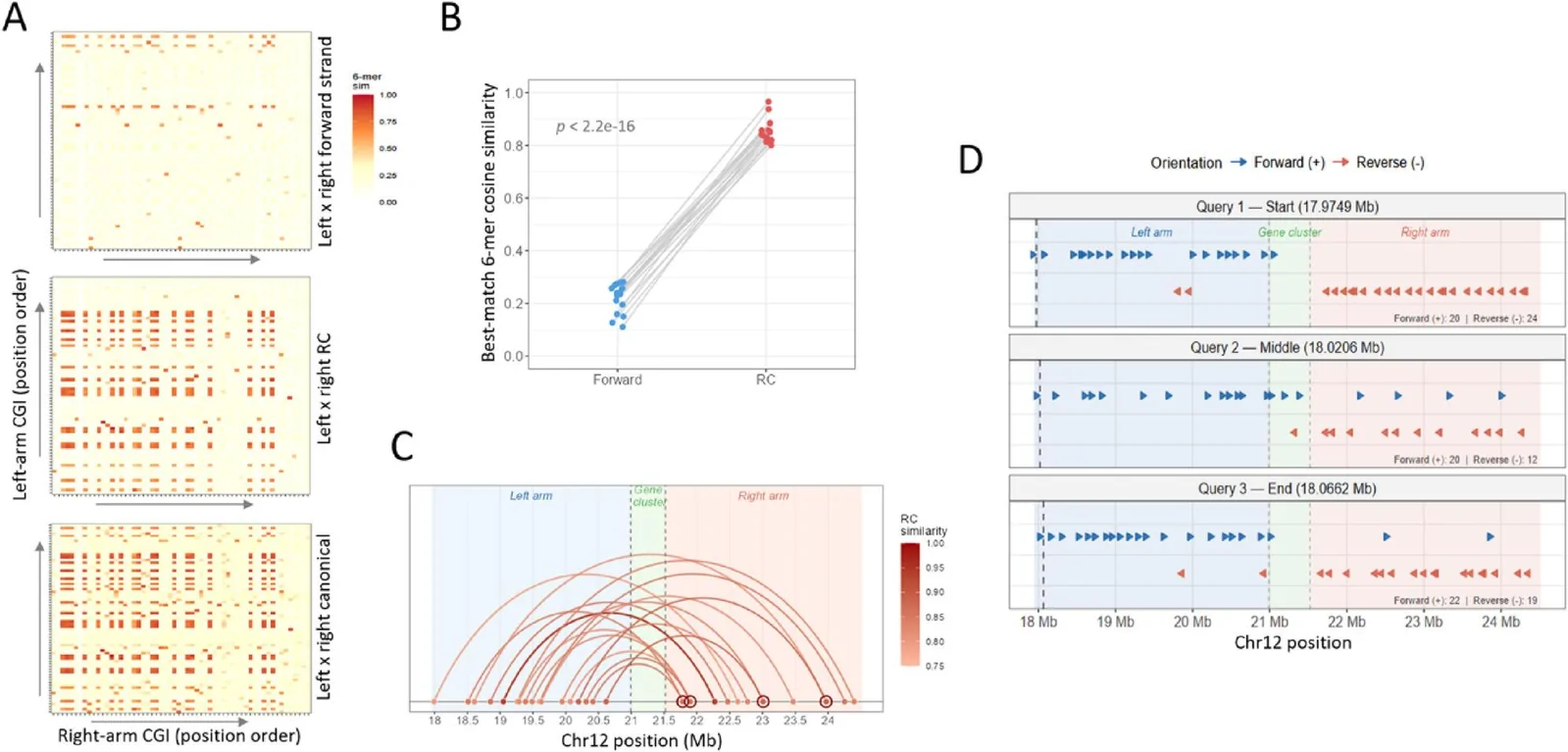
Cross-arm reverse-complement CGI similarity analysis provides sequence-level evidence for the inverted orientation of SD12A1 arms. **A** Pairwise 6-mer cosine similarity heatmaps for all left × right CGI pairs (71 × 58 = 4,118 pairs) under three comparison orientations. Left panel: forward comparison, in which left-arm CGI sequences are compared directly to right-arm CGI sequences in the standard 5′→3′ orientation (mean sim = 0.148). Middle panel: reverse-complement (RC) comparison, in which right-arm CGI sequences are reverse-complemented prior to similarity calculation (mean sim = 0.180). Right panel: canonical k-mer comparison, in which each 6-mer is replaced by the lexicographically smaller of itself and its reverse complement, removing strand information entirely (canonical vocabulary size = 2,078; mean sim = 0.276). CGIs are ordered by genomic position along both axes. Color scale indicates 6-mer cosine similarity. **B** Per-CGI best-match similarity comparison for the 18 left-arm CGIs with high-confidence reverse-complement (RC) matches (best-match RC similarity > 0.75). For each CGI, the best-match similarity against all 58 right-arm CGIs is shown in forward orientation (blue) and RC orientation (red), connected by lines. **C** Arc diagram of RC-matched CGI pairs. Each arc connects a left-arm CGI to its highest RC-similarity right-arm CGI partner, restricted to the 18 left-arm CGIs with best-match RC similarity > 0.75. Arc color reflects RC similarity (light pink to dark red). Circled right-arm CGIs indicate hub CGIs matched by two or more left-arm CGIs. **D** Directional pattern matching of three 50 bp query sequences extracted from Block 1 across the full SD12A1 locus. Queries were taken from the start (chr12:17.975 Mb), middle (chr12:18.021 Mb), and end (chr12:18.066 Mb) of Block 1 and matched against the SD12A1 sequence allowing up to five mismatches. Forward matches (blue arrows, rightward) were concentrated in the left arm, while reverse-complement matches (red arrows, leftward) were concentrated in the right arm, for all three queries. Background shading indicates arm assignment: blue = left arm, red = right arm, green = gene cluster

Given that the strand-orientation analysis established SD12A1 as an inverted palindrome, any sequence-level homology between the two arms would be expected to manifest in reverse-complement rather than forward orientation. To test this prediction directly, all left-arm and right-arm CGIs were compared using three 6-mer–based similarity measures: forward orientation, reverse-complement (RC) orientation, and strand-neutral canonical k-mers. In the forward comparison, cross-arm similarity was generally low, whereas the RC and canonical comparisons revealed a distinct subset of CGI pairs with substantially higher similarity (Fig. 4A).

For each left-arm CGI, the right-arm CGI with the highest RC-orientation similarity was identified, and analysis was focused on high-confidence matches. Applying a stringent cutoff, 18 left-arm CGIs with best-match RC similarity > 0.75 were identified. This threshold was selected empirically based on the observed distribution of best-match RC similarity values: the 18 CGIs above 0.75 form a distinct upper-tail cluster clearly separated from the bulk distribution, and no additional CGI falls between 0.75 and 0.80, indicating that this value corresponds to a natural boundary rather than an arbitrary cutoff (Supplementary Fig. S3). In all 18 cases, RC best-match similarity substantially exceeded the corresponding forward best-match similarity (RC: 0.80–0.97; forward: 0.11–0.28; Fig. 4B), confirming that sequence-level homology between these cross-arm CGI pairs is detectable specifically in the inverted orientation. These 18 CGIs were distributed broadly across the left arm, and their top-scoring right-arm partners were also spread across the right arm, consistent with preserved inverted correspondence between the two arms (Fig. 4C).

To test this inverted relationship at the level of individual duplicated sequence units, directional BLAT analysis was performed using three 50-bp queries derived from the start, middle, and end of a Block 1 repeat unit. A query length of 50 bp was chosen to balance specificity — sufficient to discriminate arm-specific hits within this repetitive locus — and sensitivity to detect homologous copies sharing ~ 93% sequence identity under a tolerance of up to 5 mismatches. Across all three queries, forward-strand hits were concentrated predominantly in the left arm, whereas reverse-strand hits were concentrated predominantly in the right arm (Fig. 4D). This directional partitioning was consistent across the repeat unit and provides independent sequence-level support that the right arm represents an inverted counterpart of the left arm rather than a direct duplication. Together, these CGI-based and sequence-level analyses confirm that homologous sequences in the two arms of SD12A1 are arranged in reverse-complement orientation, consistent with an inverted palindromic organization.

### Block-level sequence identity reveals stratified duplication history without evidence of inter-arm homogenization

**Fig. 5:**
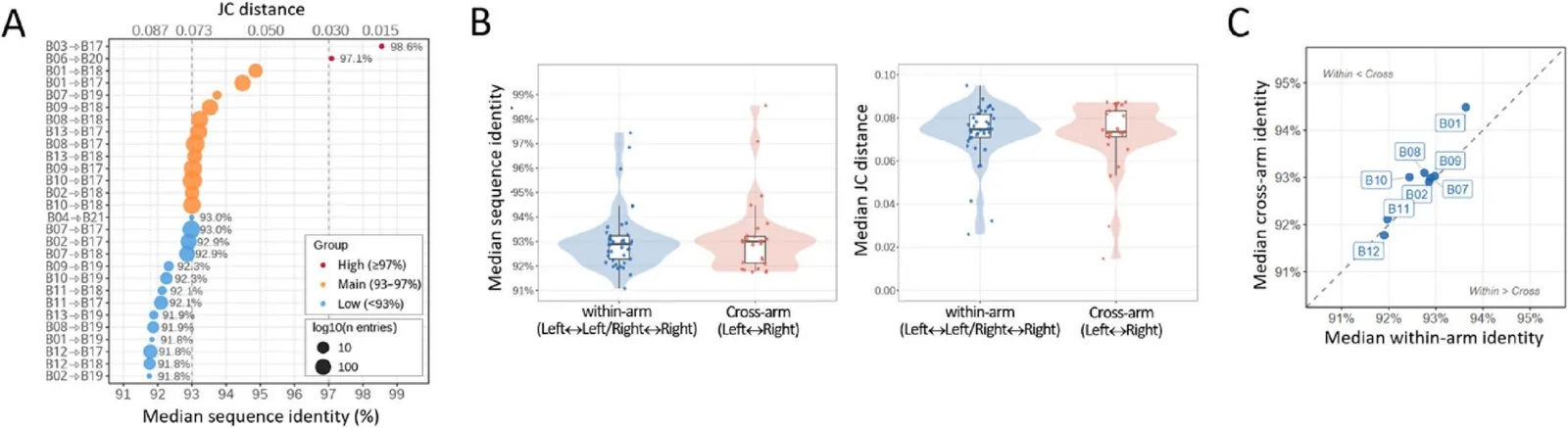
Block-level sequence identity analysis reveals stratified duplication history without evidence of inter-arm homogenization in SD12A1. **A** Cross-arm block pair sequence identity. Each point represents one unique left↔right block pair within SD12A1. Point size reflects the number of SD entries comprising each block pair (log₁₀ scale). Pairs are ranked by median pairwise sequence identity (lower x-axis; %). The upper x-axis shows the corresponding Jukes–Cantor distance. Vertical dashed lines indicate identity thresholds at 93% and 97%, defining three empirically observed groups: high-identity pairs (≥97%, red; B03↔B17 and B06↔B20), the main palindrome cluster (93–97%, orange), and lower-identity pairs (<93%, blue; B11, B12, and associated pairs). Thresholds were defined based on natural breaks in the identity distribution rather than predetermined criteria. **B** Comparison of within-arm (left↔left or right↔right; *n* = 40 pairs, blue) and cross-arm (left↔right; n = 28 pairs, red) block pair sequence identity (left) and Jukes–Cantor distance (right). Each point represents one block pair; diamond symbols indicate high-identity pairs (≥97%). Box indicates interquartile range; whiskers extend to 1.5× IQR. **C** Per-block comparison of median within-arm identity against median cross-arm identity. Each point represents one source block for which both within-arm and cross-arm pairs were available. Only 8 of 22 merged blocks are shown, as the remaining blocks either lacked within-arm pairs (single-entry blocks with no intra-arm partners) or lacked cross-arm pairs (blocks whose SD entries do not cross the gap), and were therefore excluded from the paired comparison. Block labels are shown for all plotted blocks

SD12A1 intrachromosomal SD entries showed broadly uniform cross-arm sequence identity, with most left–right block pairs clustering between 93.0% and 94.9% identity, corresponding to Jukes–Cantor (JC) distances of 0.053–0.073 (median JC = 0.0730; Fig. 5A). Within-arm and cross-arm block pairs showed nearly identical JC distance distributions (median 0.0747 vs. 0.0735, respectively; Fig. 5B), providing no evidence for differential sequence evolution between the two arms. Length-weighted mean JC distances, computed using alignment length as weight, yielded values of 0.0646 (within-arm) and 0.0650 (cross-arm), consistent with the median-based comparison and confirming that the finding of no differential sequence evolution between arms is robust to the choice of summary statistic. To assess whether this conclusion is sensitive to substitution model choice, HKY85 distances [22] were additionally computed using empirical Ti/Tv ratios derived directly from per-entry transition and transversion counts (mean Ti/Tv = 1.128); HKY85 distances were uniformly ~ 1.15× higher than JC distances across all pair types (cross-arm median: 0.0840; within-arm median: 0.0846), but the rank relationship between within-arm and cross-arm divergence was identical under both models, confirming that the finding of no differential sequence evolution between arms is robust to substitution model choice (Supplementary Fig. S4).

Two block pairs deviated markedly from this bulk distribution. B03↔B17 showed the highest cross-arm identity (98.6%, JC 0.015), involving a 4.1 kb segment whose partner lies within the large multi-entry block B17. B06↔B20 showed similarly elevated identity (97.1%, JC 0.030), representing a discrete 15 kb inverted duplication supported by a single full-length pairwise alignment (alignment length 15,002 bp of a 14,797 bp source interval). The JC distance of B06↔B20 (0.030) is less than half that of the main palindrome bulk (0.073), indicating substantially lower sequence divergence relative to the primary duplication event. In contrast, blocks B11, B12, and their associated cross-arm pairs consistently showed lower sequence identity (< 93%, JC > 0.080) relative to the palindrome bulk. Whether this reflects earlier divergence, reduced selective constraint, or local sequence features cannot be determined from pairwise identity alone.

At the per-block level, cross-arm identity equaled or exceeded within-arm identity in 7 of 8 blocks examined (mean difference, 0.24% points; Wilcoxon signed-rank test *p* = 0.055; Fig. 5C). Although this trend did not reach conventional statistical significance, likely reflecting the small number of blocks available for comparison (*n* = 8), the consistent directionality suggests that cross-arm pairs are not more diverged than within-arm pairs. Combined with the nearly identical JC distance distributions between within-arm and cross-arm comparisons (median 0.0747 vs. 0.0735; Fig. 5B), these findings provide no evidence for arm-asymmetric sequence evolution within SD12A1, and do not support ongoing gene conversion-mediated sequence homogenization comparable to that described for Y-chromosome palindromes [8, 25, 26].

### SD12A1 is the largest autosomal inverted SD cluster and a mouse-specific palindromic duplication within a broader orientation-discordant genomic interval

A genome-wide survey of large autosomal inverted SD clusters in the mouse genome was performed using the UCSC mm10 genomicSuperDups track, applying the same criteria used to define SD12A1 (intrachromosomal entries, strand = ‘−‘, fracMatch ≥ 0.90, aggregated within 2 Mb gaps). Clusters containing ≥ 100 inverted entries, a density ≥ 100 entries/Mb, and a span ≥ 2 Mb were retained (chrX and chrY excluded). This analysis identified five confirmed clusters across four autosomes, spanning 3.1–6.5 Mb with a median fracMatch of 0.930–0.946 (Supplementary Fig. S5). SD12A1 (6.5 Mb, 4,896 inverted entries) is the largest among these, exceeding the next largest candidates on chr14 (4.7 Mb and 3.8 Mb) and chr13 (3.3 Mb). The chr14 cluster shows a substantially higher entry density (1,077 entries/Mb) but a smaller span, suggesting a more compact organization relative to SD12A1.

**Fig. 6:**
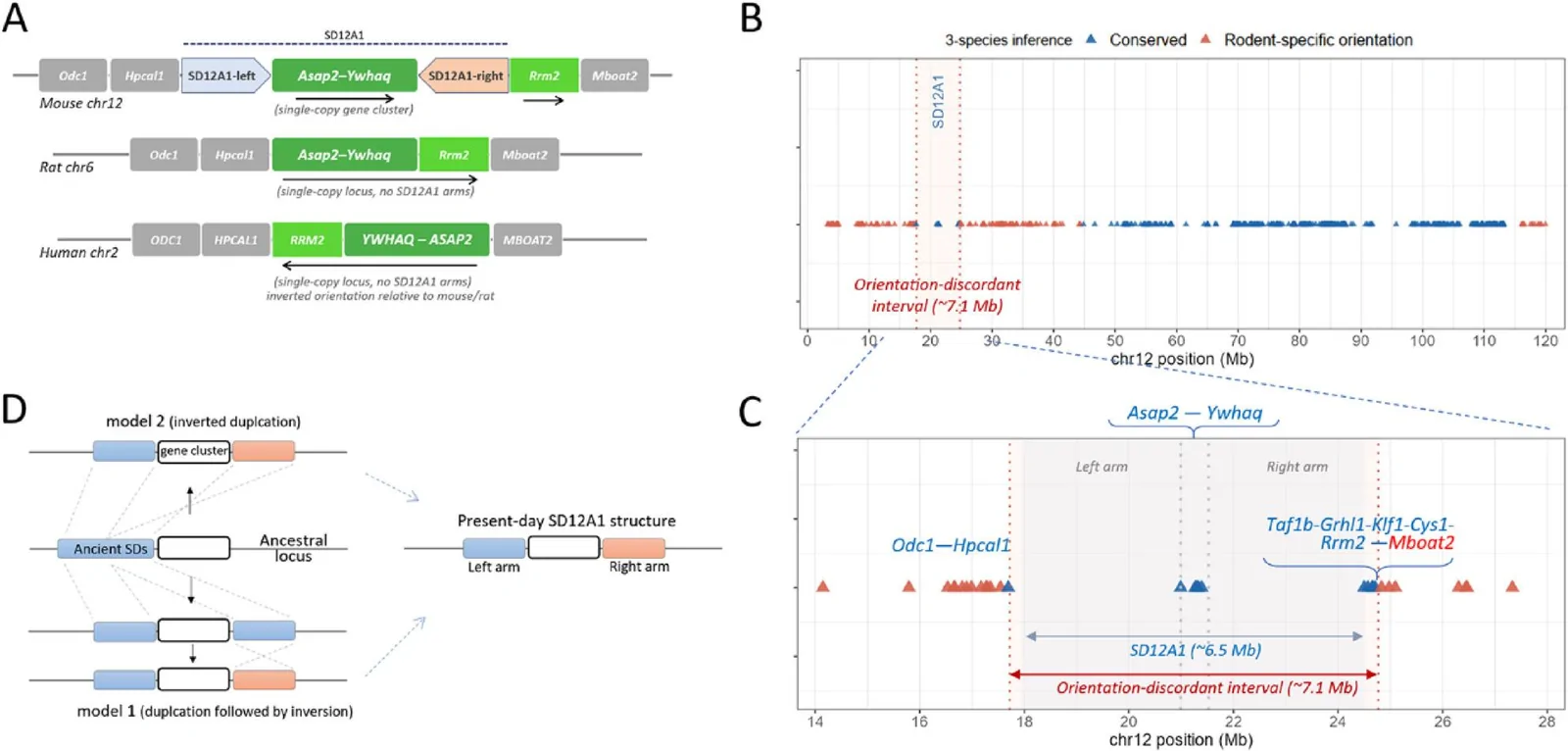
Three-species synteny analysis and evolutionary context of the SD12A1 palindrome. **A** Schematic representation of local synteny across mouse (chr12), rat (chr6), and human (chr2) at the SD12A1 orthologous interval. Gene blocks are drawn according to genomic order. In mouse, the SD12A1 palindromic arms are indicated by a blue right-pointing arrow (SD12A1-left) and an orange left-pointing arrow (SD12A1-right), flanking the single-copy Asap2–Ywhaq gene cluster (dark green). Flanking genes (Odc1, Hpcal1 proximally, and Mboat2 distally) are shown as gray blocks. Rat preserves the mouse-like local gene order but lacks the palindromic SD structure. The human interval shows the opposite genomic arrangement across this region, reflecting a rodent–human orientation discordance that predates the SD12A1 palindrome and encompasses a broader interval than SD12A1 itself (panels **B** and **C**). The Rrm2 locus (light green), although located outside SD12A1, participates in the same rodent–human orientation-discordant arrangement, indicating that the broader rearranged orthologous configuration extends beyond the palindromic duplication itself. **B**-**C** Whole-chromosome (**B**) and regional (C, chr12:14–28 Mb) views of transcriptional-orientation relationships inferred from three-species comparison of mouse, rat, and human orthologs. Each triangle represents one orthologous gene comparison. Blue triangles denote genes with conserved transcriptional orientation across all three species, whereas red triangles denote genes for which mouse and rat share the same orientation but are discordant with the human ortholog. The orientation-discordant interval (~7.1 Mb, salmon shading in C and red dotted boundaries in **B** and **C**) encompasses the entire SD12A1 region. In panel **C**, the blue horizontal bar marks the SD12A1 interval (~6.5 Mb), and gray dashed vertical lines indicate the central gap boundaries. The proximal boundary of the orientation-discordant interval falls between Hpcal1 and Asap2 (~17.7 Mb), and the distal boundary falls between Rrm2 and Mboat2 (~24.8 Mb), as indicated by the red dotted lines. The central gap gene cluster (Asap2–Itgb1bp1–Cpsf3–Iah1–Adam17–Ywhaq) is indicated by the blue bracket above panel **C**. **D** Schematic models for the possible origin of the present-day SD12A1 structure. In Model 1, pre-existing ancestral SD sequence is first distributed to both sides of the ancestral locus, and a subsequent inversion reorients one flanking segment, generating oppositely oriented arms. In Model 2, an inverted duplication generates the same overall configuration in a single step. Both models can account for the present-day SD12A1 structure, in which the central gene-containing spacer is flanked by left and right SD-rich arms arranged in opposite orientation. Blue and orange blocks denote duplicated arm segments in opposite orientations. Black arrows indicate conceptual rearrangement steps, blue dashed arrows indicate convergence on the present-day structure, and gray dashed lines indicate conceptual correspondence among segments rather than exact sequence mapping. The schematic therefore supports the final inverted organization but does not resolve the temporal order of duplication and inversion

The *Asap2–Ywhaq* gene cluster is conserved as a single-copy locus in the corresponding human chromosome 2 interval (hg38, chr2:9.2–9.6 Mb) and rat chromosome 6 interval (rn7, chr6:39.5–41.5 Mb), and neither species contains SD12A1-like flanking segmental duplications or palindromic architecture at this locus (Fig. 6A). Thus, the palindromic SD structure itself appears to have arisen specifically in the mouse lineage after the mouse–rat divergence. Despite the mouse-specific nature of the SD12A1 duplication, three-species comparison of the transcriptional orientation of orthologous genes identified a broader 7.1 Mb orientation-discordant orthologous interval in which mouse and rat share gene orientations that differ from their human orthologs (Fig. 6B and C). Across 4,837 orthologous gene comparisons on mouse chr12, the orientation patterns resolved into two classes: 2,321 genes (48%) showed rodent-shared discordance relative to human, whereas 2,516 genes (52%) were concordant across all three species. This orientation-discordance analysis was informative because it delimited the broader rearranged orthologous interval in which SD12A1 is embedded. The proximal boundary was localized between *Hpcal1* and *Asap2* (~ 17.7 Mb), and the distal boundary between *Rrm2* and *Mboat2* (~ 24.8 Mb; Fig. 6C). The SD12A1 palindrome falls entirely within this interval. Importantly, *Rrm2* lies outside SD12A1 but participates in the same rodent–human orientation-discordant arrangement, indicating that the broader rearranged configuration extends beyond the palindromic duplication itself. These observations support a model in which the mouse-specific SD12A1 palindrome arose within a pre-existing orientation-discordant orthologous interval, rather than creating the entire rodent–human orientation difference. However, the polarity of the ancestral orientation, and therefore the lineage in which the broader orientation difference arose, cannot be resolved without additional outgroup species.

## Discussion

Collectively, these analyses support interpreting SD12A1 as a large inverted palindrome composed of two SD-rich arms flanking a gene-containing central spacer. This conclusion rests on convergent evidence from several independent analytical layers: inter-arm SD pairs are predominantly inverted whereas intra-arm pairs are predominantly direct; short query sequences map in opposite strand orientations to the two arms; a subset of cross-arm CGI pairs shows strong reverse-complement similarity; and three-species synteny places the locus within a broader orientation-discordant interval. Together with the broadly similar divergence profiles of the two arms, these features argue for a common duplication ancestry rather than two unrelated repeat clusters, and they illustrate an analytical framework that could be applied to candidate palindromic intervals in other species or genome assemblies.

A parsimonious formation model is that ancestral SD sequences adjacent to the *Asap2*–*Ywhaq* interval were duplicated or redistributed and subsequently rearranged in the mouse lineage, leaving the gene-containing interval as the spacer between two oppositely oriented SD-rich arms (Fig. 6D). However, the present reference-based analysis cannot resolve the exact source sequence, fine-scale breakpoint structure, strain-level copy-number variation, or temporal order of duplication and inversion events. These limitations are not merely technical. The order of events bears directly on the mechanistic model: a sequential duplication-then-inversion process, a rapid rearrangement of pre-existing SD sequences, and a single-step inverted duplication imply different mutational pathways and potentially different recombination or replication intermediates. Long-read assemblies across multiple mouse strains, combined with additional comparative outgroup analyses, will therefore be required to reconstruct the formation history of SD12A1. Whether these locally asymmetric subregions correspond to structural-variation hotspots remains to be tested using strain-resolved long-read assemblies or population-scale WGS data.

The central gap containing *Asap2–Ywhaq* is gene-rich and largely depleted of SD entries, making it a natural structural spacer. The CGI-based axis estimates (mean 21.11 Mb, median 21.10 Mb, SD 0.57 Mb, *n* = 18 high-confidence pairs) place the implied inversion axis within the gap, broadly consistent with the gap center (21.26 Mb), with no statistically meaningful directional offset. Consistently, the arc plot connecting each high-confidence left-arm CGI to its best RC-matched right-arm counterpart shows visually symmetric arc spans across the gap, with left- and right-arm anchor points distributed at comparable distances from the gap boundary (Fig. 4C). The gap is therefore interpreted as a functional spacer whose geometric center approximates the inversion axis, though the large spread of individual estimates (SD = 0.57 Mb) reflects residual heterogeneity in cross-arm CGI pairing that prevents a precise determination of the inversion boundary.

Cross-species comparison places SD12A1 within a larger orientation-discordant orthologous interval (~ 17.7–24.8 Mb) shared by mouse and rat relative to human, although the lineage in which this polarity difference arose cannot be resolved without additional outgroup species. The fact that this interval fully encompasses the mouse-specific SD12A1 palindrome suggests that the palindrome arose within a pre-existing rearranged genomic context rather than at an arbitrary genomic position. This interpretation is consistent with the broader association between SDs and evolutionary rearrangement breakpoints across mammalian genomes [27–29]. At the present locus, three observations converge on this view: the mouse-specific palindrome, the broader rodent–human orientation discordance, and recurrent pathogenic inverted duplications reported at the human orthologous *ASAP2*–*HPCAL1* region [16–18]. Together, these observations suggest that the *Asap2*–*Ywhaq* interval has sustained independent structural changes across mammalian lineages. This pattern is consistent with a fragile-site model in which local genomic features, including elevated SD density [27, 30, 31], non-B DNA-forming potential [32, 33], or replication fork instability [34, 35], predispose specific intervals to recurrent large-scale rearrangement. Whether the same underlying properties contributed both to mouse palindrome formation and to human pathogenic inversions or duplications remains an important mechanistic question. Thus, SD12A1 is best interpreted not as an isolated anomaly, but as a mouse-specific palindromic manifestation of recurrent structural instability at an evolutionarily labile genomic interval centered on the *Asap2*–*Ywhaq* gene cluster.

SD12A1 shares the defining structural features of palindromic SD architecture with the palindromes characterized on the human Y chromosome and at several autosomal loci. However, it differs from each of these precedents in ways that illuminate the structural and evolutionary diversity of palindromic organization across mammalian genomes. The Y-chromosome palindromes (P1–P8) represent the most extensively studied examples of this architecture, where arm-to-arm sequence identity exceeding 99.9% is actively maintained by inter-arm gene conversion [8, 26]. This gene conversion is itself understood as a consequence of selective pressure to preserve the multiple functional gene copies — including *DAZ*, *CDY*, and *HSFY* — carried within the palindrome arms [8]. SD12A1 presents a fundamentally different configuration: its arms are dominated by duplicated noncoding or fragmentary sequence rather than annotated dosage-sensitive gene families, and the annotated functional genes are concentrated in the spacer rather than carried as functional duplicates within both arms. This structural distinction reduces any obvious selective rationale for maintaining near-perfect arm-to-arm sequence identity, providing a parsimonious explanation for the absence of gene conversion evidence and the substantially lower cross-arm identity (~ 93%) observed in SD12A1. In this sense, SD12A1 represents a palindromic SD locus in which the two arms have been evolving independently under neutral drift since the initial duplication event, contrasting sharply with the actively homogenized Y-chromosome palindromes.

Beyond the canonical Y-chromosome examples, large inverted-repeat architectures have also been described elsewhere in the human genome. In particular, Warburton et al. (2004) reported that the human X chromosome contains a disproportionate number of large, highly homologous inverted repeats, many of which include testis-associated genes [36]. Human autosomal loci provide additional but non-equivalent precedents for rearrangement-prone palindromic or inverted duplicated architecture. *GOLGA8*-based palindromic core duplicons at 15q13.3 represent an SD-related autosomal example associated with recurrent microdeletion breakpoints and evolutionary instability [9]. This locus is relevant to the general concept that palindromic duplicon organization can promote recurrent rearrangement, although it differs from SD12A1 in scale, lineage context, and internal organization. By contrast, short AT-rich palindromes such as those at 22q11.2 [37] are better viewed as non-B DNA-forming rearrangement substrates rather than direct structural analogs of the megabase-scale head-to-head SD architecture described here. In this context, SD12A1 expands the known repertoire of mammalian palindromic architectures by showing that a large inverted SD-rich structure can arise on a mouse autosome outside the canonical Y-chromosome setting.

## Conclusions

SD12A1 represents a previously undescribed large-scale autosomal palindromic SD architecture in the mouse genome, extending the known diversity of palindromic segmental duplications beyond the canonical Y-chromosome context in which such structures were first characterized. Its identification shows that palindromic SD organization is not restricted to sex chromosomes or to loci maintained by strong selective pressure for inter-arm gene conversion, but can also arise on autosomes where the two arms evolve largely independently. Because SD12A1 lies within an evolutionarily labile interval that is also rearrangement-prone in the human ortholog, it provides both a concrete structural exemplar and a tractable model locus for studying how repetitive genomic architecture shapes chromosome evolution and mutational vulnerability. Future long-read assemblies across diverse mouse strains, together with broader mammalian outgroup comparisons, will be needed to resolve the fine-scale breakpoint structure, determine whether SD12A1 is fixed or polymorphic within Mus musculus, and reconstruct the sequence of events that generated this large palindromic architecture.

## Supplementary Information

Supplementary Material 1.

## Data Availability

No new sequencing or other primary experimental datasets were generated in this study. Publicly available genomic sequence and annotation data used for the analyses were obtained from the UCSC Genome Browser resources for the mouse mm10/GRCm38 and mm39/GRCm39 assemblies, including genomic sequence, segmental duplication, RepeatMasker, gene annotation, and CpG island annotation tracks. The derived data supporting the conclusions of this article are included in the article and its supplementary information files. Additional intermediate files and scripts used for coordinate processing and figure generation are available from the corresponding author upon reasonable request.

## Abbreviations

CGI: CpG island
JC: Jukes–Cantor
NAHR: Non-allelic homologous recombination
RC: Reverse complement
SD: Segmental duplication
SD12A1: Segmental duplication in chr12 qA1 region
UCSC: University of California, Santa Cruz
