# Supplementary material for: A large palindromic segmental duplication on mouse chromosome 12 marks an evolutionarily fragile genomic interval
Source: BMC Genomics. 2026 Jul 27;27:877. doi: 10.1186/s12864-026-13210-y (PMC13688818; doi:10.1186/s12864-026-13210-y)
Supplement: Supplementary file 1 [file 12864_2026_13210_MOESM1_ESM.pdf]

Supplementary Fig. S1

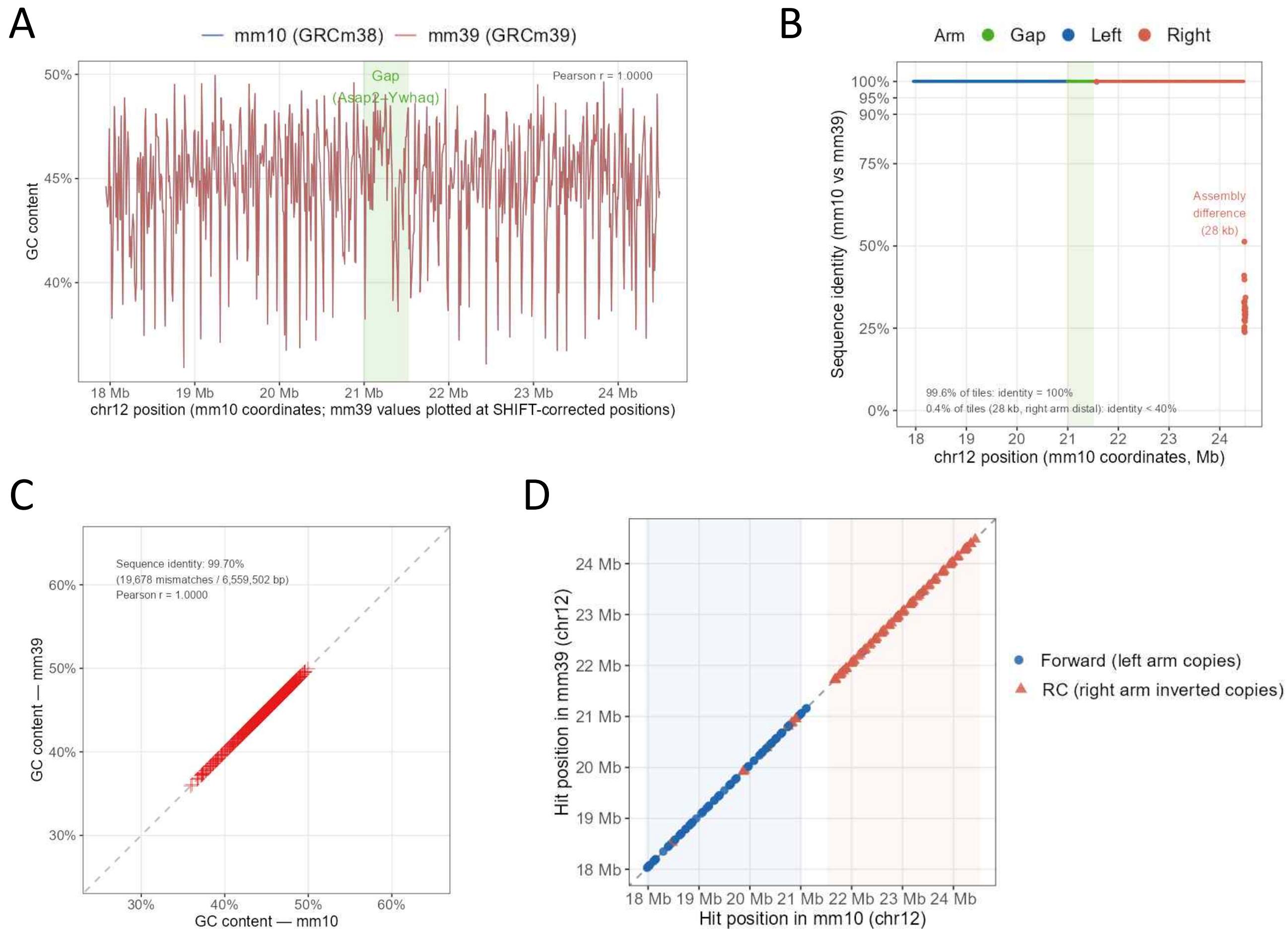

Supplementary Fig. S1. Cross-assembly validation of SD12A1 between mm10 (GRCm38) and mm39 (GRCm39). (A) GC-content profiles of the SD12A1 interval in mm10 (chr12:17.95–24.51 Mb; blue) and the corresponding mm39 orthologous region (chr12:18.00–24.56 Mb; red), computed in non-overlapping 10 kb tiles. mm39 values are plotted at shift-corrected mm10 coordinates (shift = +50,001 bp), anchored on Asap2 exon 1. The two profiles are visually indistinguishable (Pearson r = 1.000, n = 655 tiles). Green shading indicates the central gap region containing the Asap2–Ywhaq gene cluster (chr12:20.99–21.52 Mb). (B) Tile-by-tile sequence identity between mm10 and mm39 at 1 kb resolution across the SD12A1 locus (n = 6,559 tiles). Points are colored by arm assignment: left arm (blue), gap (green), and right arm (red). Of 6,559 tiles, 6,530 (99.6%) showed perfect sequence identity (100%) between assemblies. Among the remaining 29 tiles, 28 contiguous tiles spanning a 28 kb interval at the distal boundary of the right arm (chr12:24.4815–24.5085 Mb, mm10; salmon shading) showed reduced identity (23.8–51.3%), in a region outside the palindromic core structure. One additional isolated tile near the central gap (chr12:21.5705 Mb) showed a single mismatch (99.9% identity). The right-arm boundary divergence may reflect assembly updates between GRCm38 and GRCm39. (C) Per-tile GC-content concordance between mm10 and mm39, plotting GC content in each 10 kb tile in mm10 against the corresponding shift-corrected mm39 tile. GC-content values fell tightly along the identity line (dashed gray; y = x; Pearson r = 1.0000), confirming concordant base composition between assemblies across the locus. (D) Scatter plot of left-arm seed hit positions in mm10 (x-axis) versus mm39 (y-axis). Eight 100 bp seeds were extracted from confirmed left-arm SD block intervals and searched against both assemblies (≤10 mismatches). Forward hits (blue circles) map to left-arm positions; reverse-complement hits (red triangles) map to right-arm positions, reflecting the inverted relationship between arms. The dashed gray line indicates the expected coordinate shift (y = x + 50,001 bp). Forward and reverse-complement hit counts were identical between mm10 and mm39 for all eight seeds, confirming that the palindromic arm structure is preserved in mm39.

Supplementary Fig. S2

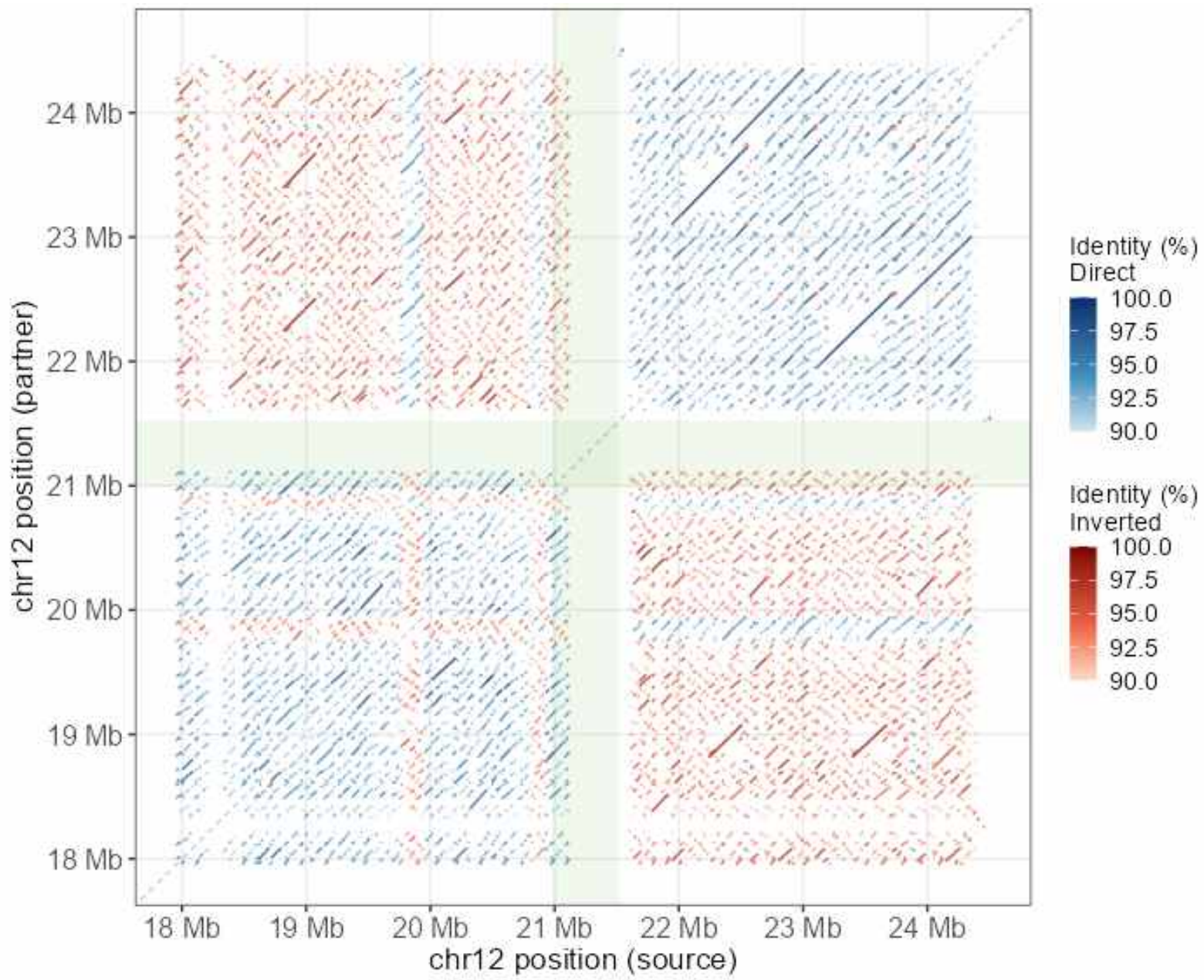

Supplementary Fig. S2. StainedGlass-style self-alignment dot plot of SD12A1. Each segment represents one UCSC mm10 genomicSuperDups intrachromosomal pairwise SD entry within SD12A1, plotted according to its source and partner coordinates. Blue segments indicate direct-orientation alignments, and red-to-orange segments indicate inverted-orientation alignments. Color intensity represents sequence identity within the 90–100% range covered by the genomicSuperDups annotation. Green shading marks the SD-depleted central gap containing the Asap2–Ywhaq gene cluster. The plot confirms the quadrant structure shown in Fig. 3A, with direct-orientation relationships enriched within arms and inverted-orientation relationships predominating across arms.

Supplementary Fig. S3

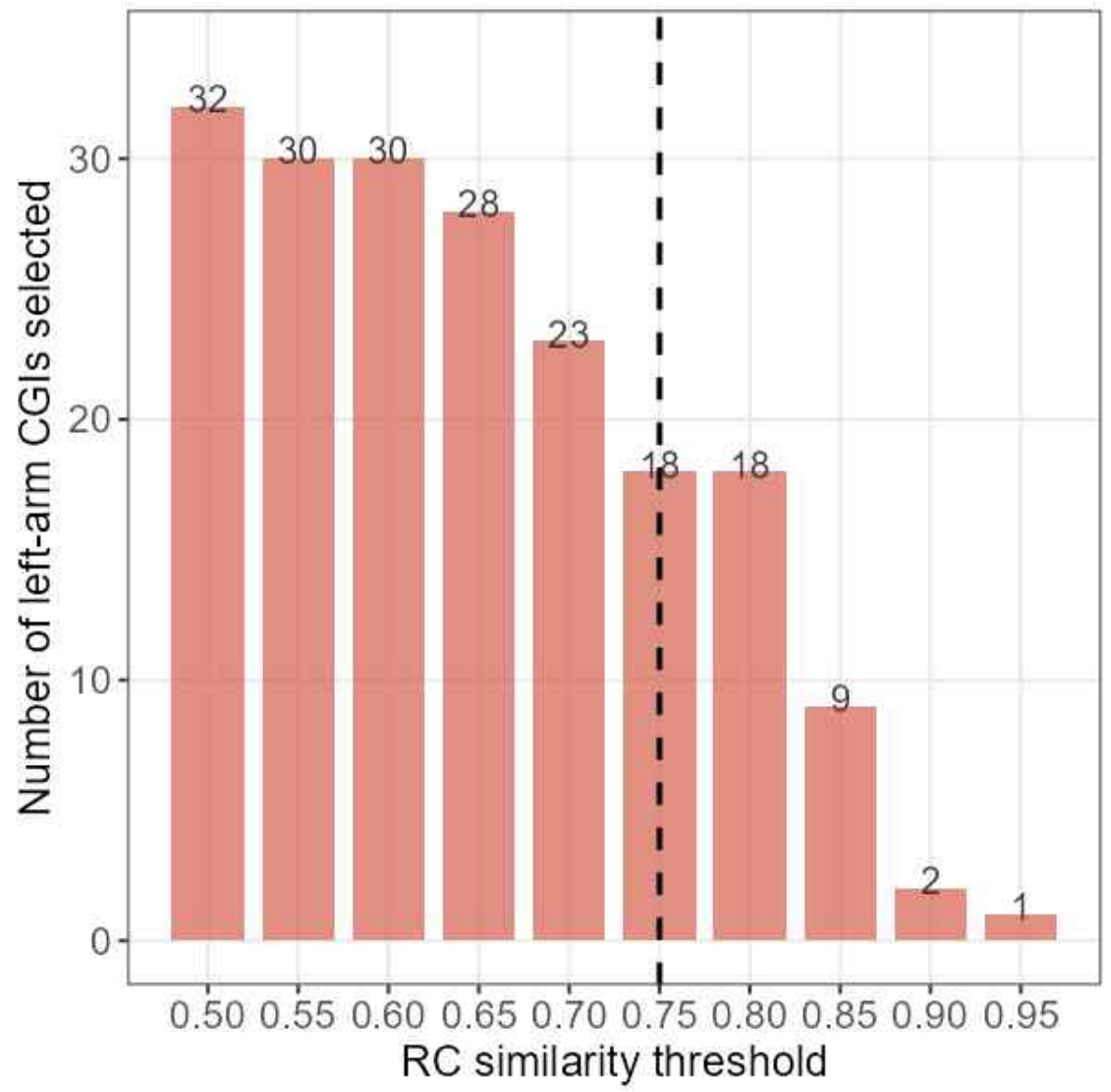

Supplementary Fig. S3. Justification of the reverse-complement (RC) cosine similarity threshold (>0.75) for high-confidence cross-arm CGI pair identification. Sensitivity analysis showing the number of left-arm CGIs selected at RC similarity thresholds ranging from 0.50 to 0.95. The number of selected CGIs is identical at thresholds of 0.75 and 0.80 (18 CGIs), indicating that 0.75 corresponds to a natural gap in the distribution rather than an arbitrary boundary. Lowering the threshold to 0.70 adds five CGIs with a substantially smaller mean RC–forward gap (0.491 vs. 0.562 at 0.75), indicating that these additional cases are less unambiguous.

Supplementary Fig. S4

A

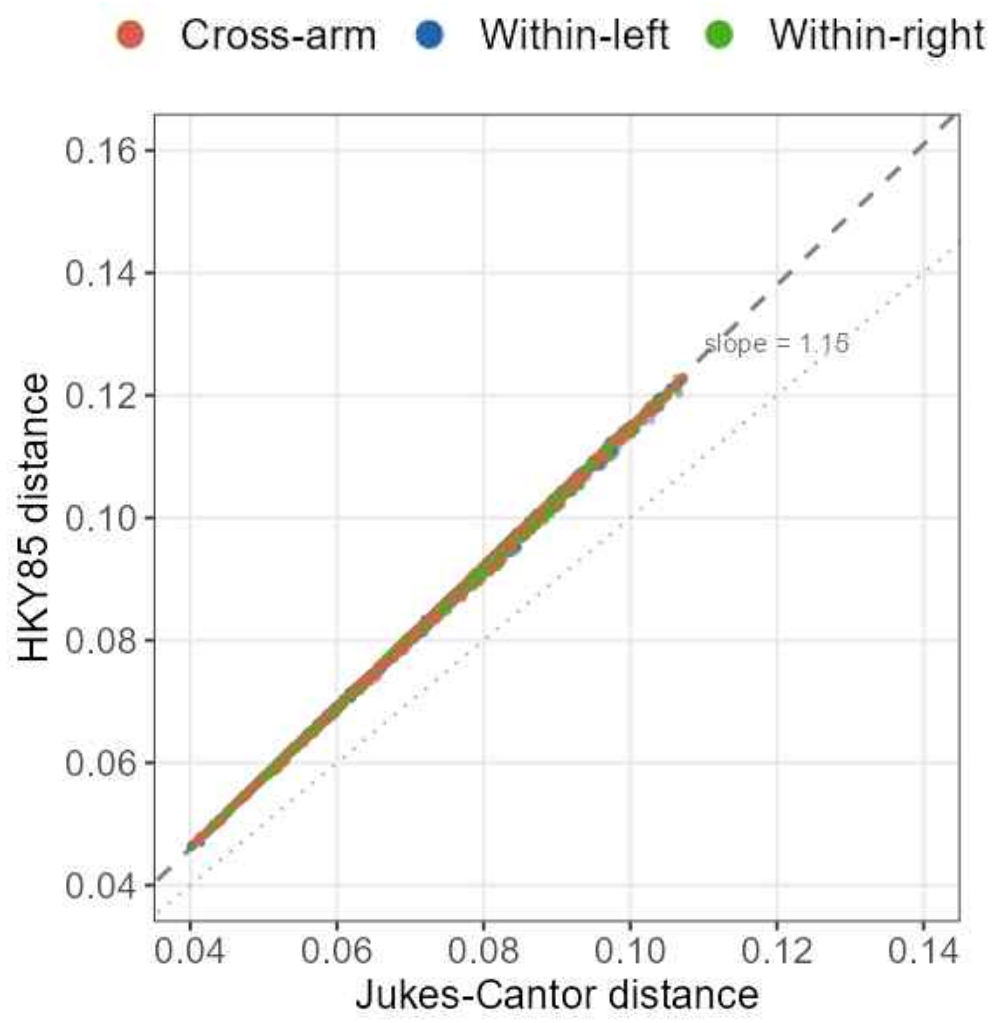

B

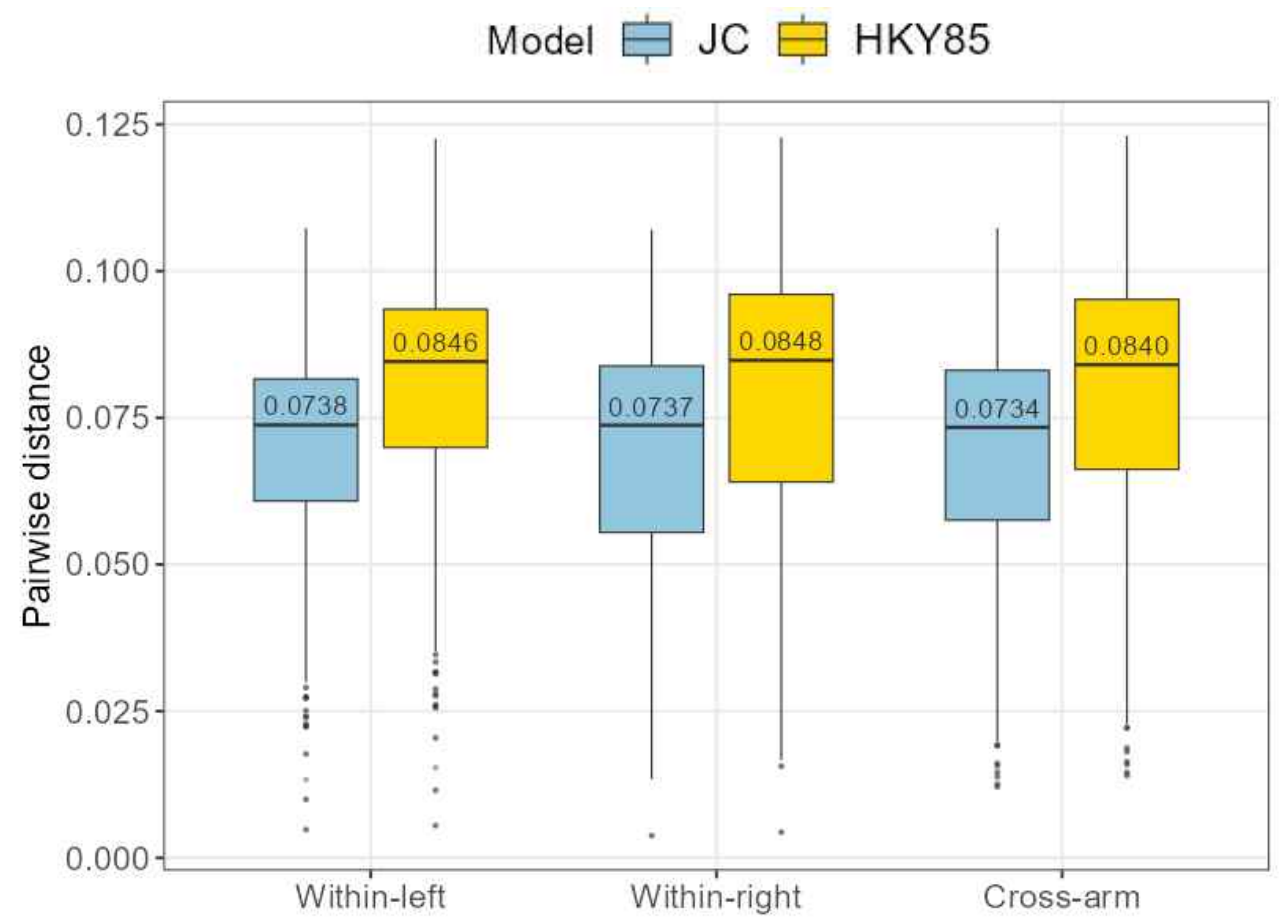

Supplementary Fig. S4. Comparison of Jukes–Cantor (JC) and HKY85 substitution-model distances for SD12A1 intrachromosomal segmental-duplication entries. (A) Scatter plot of JC distance (x-axis) versus HKY85 distance (y-axis) for all SD12A1 intrachromosomal SD entries (n = 8,853), colored by pair type: cross-arm (red), within-left (blue), and within-right (green). The dashed line indicates a slope of 1.15, and the dotted line indicates slope = 1 (identity). Points from all three pair types overlap almost completely along the slope-1.15 line, indicating that HKY85 distances are uniformly approximately 1.15-fold higher than JC distances regardless of pair type. HKY85 distances were computed using empirical Ti/Tv ratios derived from per-entry transition and transversion counts in the UCSC genomicSuperDups track (mean Ti/Tv = 1.128). (B) Boxplots of pairwise distance distributions by pair type (within-left, within-right, cross-arm) and substitution model (JC: blue; HKY85: red). Median values are indicated within each box. The rank relationship between within-arm and cross-arm distances is identical under both models (within-arm median: JC = 0.0737–0.0738, HKY85 = 0.0846–0.0848; cross-arm median: JC = 0.0734, HKY85 = 0.0840), confirming that the finding of no differential sequence evolution between arms is robust to substitution-model choice.

Supplementary Fig. S5

A

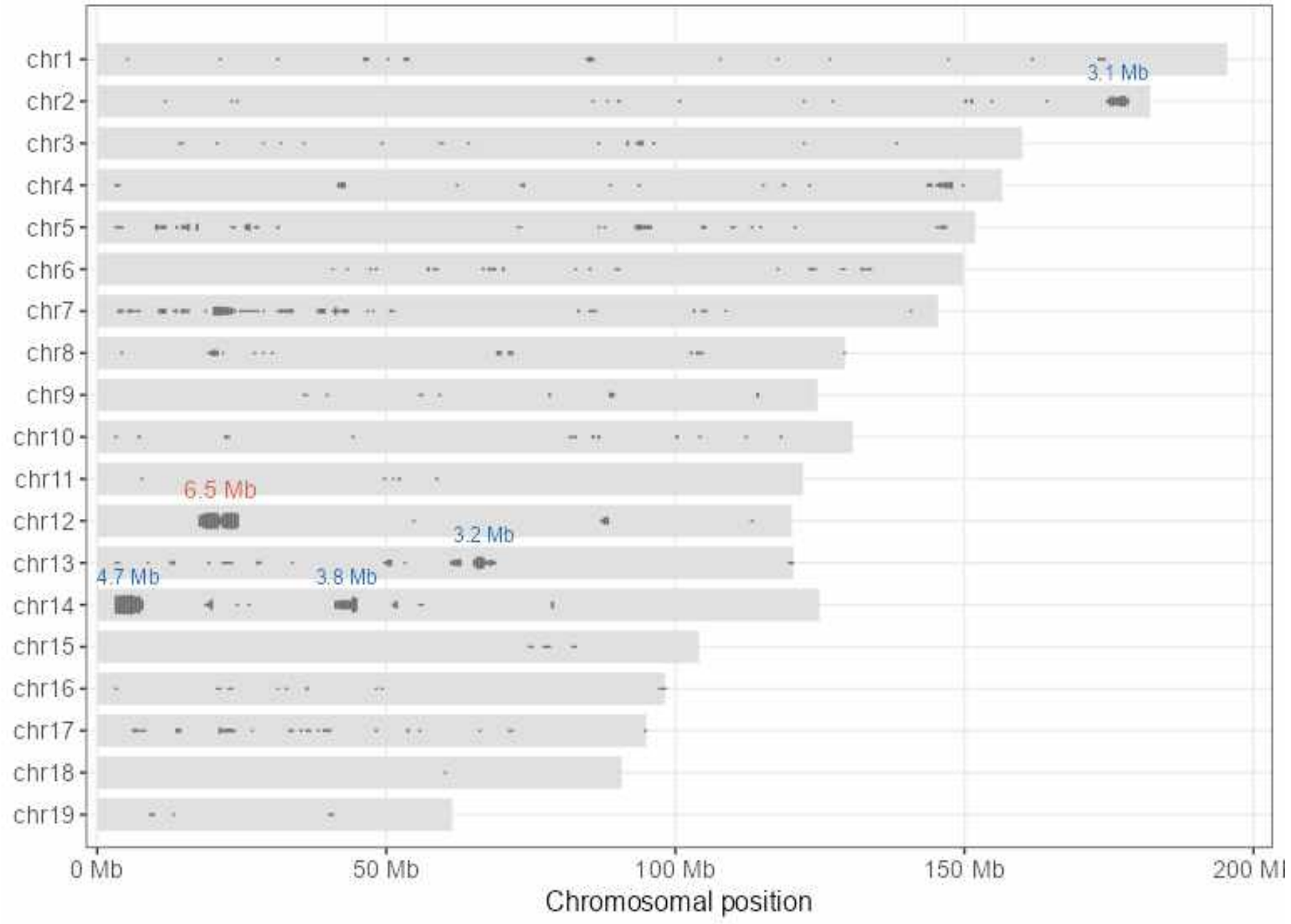

B

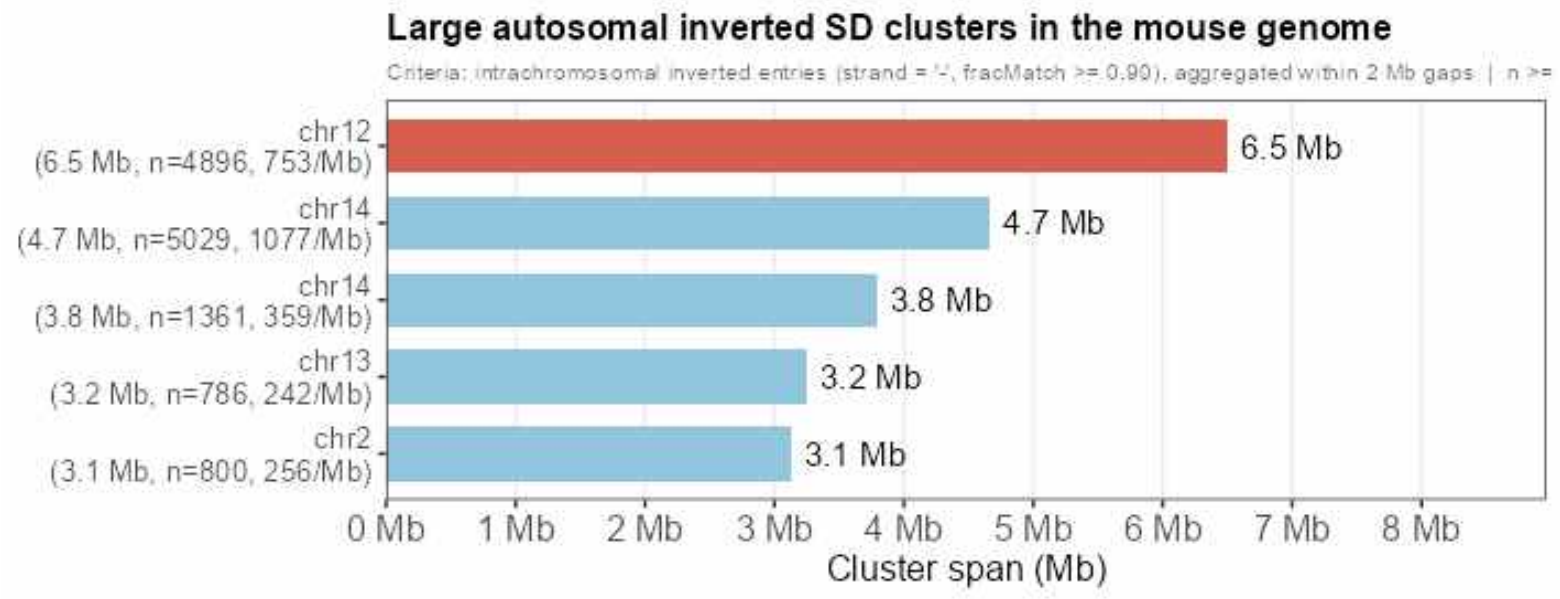

C

Chr	Start	End	No. inverted entries	Span (Mb)	Center (Mb)	Density (entries/Mb)	Median fracMatch
chr12	17,950,503	24,510,004	4,896	6.5	21.1	753	0.930
chr14	3,000,000	7,670,248	5,029	4.7	5.3	1,077	0.933
chr14	41,202,673	44,996,041	1,361	3.8	43.1	359	0.943
chr13	65,309,042	68,562,845	786	3.3	66.9	242	0.937
chr2	174,958,961	178,089,707	800	3.1	177.0	256	0.946

Supplementary Fig. S5. Genome-wide survey of large autosomal inverted segmental-duplication clusters in the mouse genome (mm10). (A) Chromosome-wide map showing the distribution of autosomal inverted SD entries across all 19 mouse autosomes. Gray marks indicate 500 kb bins containing inverted SD entries (fracMatch ≥ 0.90, strand = '−'), with darker or thicker marks representing higher entry counts. SD12A1 (chr12, red label) and other confirmed large clusters (blue labels) are annotated with their genomic spans. The large span and high entry density of SD12A1 relative to other autosomal loci are visually apparent. (B) Bar chart showing the genomic span of confirmed large autosomal inverted SD clusters. Clusters were identified from the UCSC mm10 genomicSuperDups track using the same criteria applied to define SD12A1: intrachromosomal entries with strand = '−' and fracMatch ≥ 0.90 were aggregated within 2 Mb gaps, and clusters were retained if they contained ≥100 inverted entries and showed a density ≥100 entries/Mb; chrX and chrY were excluded. Five clusters meeting these criteria were identified across four autosomes. SD12A1 (chr12, red) is the largest cluster, with a span of 6.5 Mb; the remaining four clusters (chr14, chr14, chr13, chr2) span 3.1–4.7 Mb (blue). Labels indicate chromosome, cluster span, number of inverted entries, and entry density. (C) Summary table of confirmed large autosomal inverted SD clusters. Clusters are listed with genomic coordinates (mm10), number of inverted SD entries, cluster span, chromosomal center position, entry density, and median fracMatch, ordered by decreasing span. The chr12 cluster corresponds to SD12A1; coordinates reflect the full SD12A1 interval (chr12:17,950,503–24,510,004) as defined in the primary analysis. The remaining clusters are identified solely from their UCSC genomicSuperDups entry distributions and have not been subjected to sequence-level palindromic validation. fracMatch values reflect the median pairwise sequence identity between source and partner sequences as reported in the UCSC genomicSuperDups track.
